# New high-sensitivity troponin test reduces heart attack risk

**Published:** 2018

The newer high-sensitivity troponin test discovers smaller amounts of heart-specific proteins, troponins, than the older troponin test and therefore identifies more myocardial infarction patients than before. A study from Karolinska Institutet in Sweden now reports that the risk of a future heart attack is lower in patients diagnosed with the new test.

A blood test that measures the presence of heart-specific proteins called troponins is used by emergency clinics to diagnose myocardial infarction in patients with chest pain.

For the past few years a newer laboratory method has been used at most hospitals in Sweden that is 10n times more sensitive than the conventional troponin test. The high-sensitivity troponin test can discover heart attacks earlier so that treatment can commence, which is thought to improve the patient’s prognosis. ‘But there is a lack of larger studies examining whether the high-sensitivity troponin test is of any significance for patients with newly diagnosed myocardial infarction in terms of survival or the risk of another heart attack,’ says study leader Dr Martin Holzmann, associate professor of epidemiology at Karolinska Institutet’s Department of Medicine in Solna and physician at Karolinska University Hospital.

The study included all patients in Sweden who had had their first heart attack between 2009 and 2013. This gave a study population of almost 88 000 patients, 40 000 of whom had been diagnosed using the high-sensitivity troponin test and just over 47 000 using the conventional troponin test.

The researchers found that 5% more myocardial infarctions were being diagnosed in hospitals that used the high-sensitivity troponin test. A year after the heart attack was registered there was no difference in mortality rate between the two groups, although the number of new heart attacks was lower in the group that had been diagnosed using the high-sensitivity troponin test.

‘This surprised us,’ says Holzmann. ‘We didn’t think that the more sensitive test would affect the risk of future heart attacks.’

The use of coronary angiography and balloon angioplasty was 16 and 13% more common, respectively, in the patients diagnosed with the high-sensitivity troponin test. In the USA, where the new test was not approved until 2017, there are fears that the more sensitive methods can entail a large increase in the number of examinations with no benefit to the patients.

‘The increase we observed in our study was less than expected, which means that the high-sensitivity troponin test has enabled doctors to single out the patients who benefit from such intervention. We found no differences in medication between the two groups, so the differences in prognosis with fewer new heart attacks could be attributed to the fact that more coronary angiography and balloon dilation procedures have been performed on the right patients,’ says Holzmann, who also believes that the study supports the idea that the handful of hospitals in Sweden that still do not use the high-sensitivity troponin test should start to do so.
